# Prophylactic immunization to *Helicobacter pylori* infection using spore vectored vaccines

**DOI:** 10.1111/hel.12997

**Published:** 2023-06-14

**Authors:** Paidamoyo M. Katsande, Van Duy Nguyen, Thi Lan Phuong Nguyen, Thi Kim Cuc Nguyen, Gabrielle Mills, David M. D. Bailey, Graham Christie, Huynh Anh Hong, Simon M. Cutting

**Affiliations:** ^1^ Department of Biological Sciences Royal Holloway University of London Egham UK; ^2^ Institute of Biotechnology and Environment Nha Trang University Nha Trang Vietnam; ^3^ Institute of Vaccines and Biological Medicals (IVAC) Nha Trang Vietnam; ^4^ Department of Chemical Engineering & Biotechnology University of Cambridge Cambridge UK

**Keywords:** *Helicobacter pylori* infection, oral and parenteral immunization, vaccine

## Abstract

**Background:**

*Helicobacter pylori* infection remains a major public health threat leading to gastrointestinal illness and increased risk of gastric cancer. Mostly affecting populations in developing countries no vaccines are yet available and the disease is controlled by antimicrobials which, in turn, are driving the emergence of AMR.

**Materials and Methods:**

We have engineered spores of *Bacillus subtilis* to display putative *H. pylori* protective antigens, urease subunit A (UreA) and subunit B (UreB) on the spore surface. Following oral dosing of mice with these spores, we evaluated immunity and colonization in animals challenged with *H. pylori*.

**Results:**

Oral immunization with spores expressing either UreA or UreB showed antigen‐specific mucosal responses (fecal sIgA) including seroconversion and hyperimmunity. Following challenge, colonization by *H. pylori* was significantly reduced by up to 1‐log.

**Conclusions:**

This study demonstrates the utility of bacterial spores for mucosal vaccination to *H. pylori* infection. The heat stability and robustness of *Bacillus* spores coupled with their existing use as probiotics make them an attractive solution for either protection against *H. pylori* infection or potentially for therapy and control of active infection.

## 1 INTRODUCTION

*Helicobacter pylori* (HP) is a Gram‐negative human pathogenic bacterium believed to be carried by an estimated 50% of the world's population.^1^ Infection typically occurs in childhood and if untreated an individual will likely remain colonized for life.^2^ Of those infected about 15% may go on to develop pathological symptoms, typically chronic gastritis and peptic ulcer disease (gastric and duodenal ulceration) with the potential for gastric cancer (adenocarcinoma and lymphoma). *H. pylori* infection is thought to be linked with more than 90% of gastric cancers and normally results if the infection is left untreated.^3^ Gastric adenocarcinoma is now the 5th most common cancer and the 3rd in terms of cancer‐related deaths. Unfortunately, by the time gastric cancer has developed it is nearly impossible to eradicate the underlying infection. Humans appear to acquire *H. pylori* in two ways, oral‐oral being the most common route of transmission and vertical transmission from mother to child.^2^ *H. pylori* is unusual in that it is able to colonize the stomach where this highly motile bacterium can penetrate mucus, cause inflammation, and degrade the stomach lining. Incidence rates appear higher in developing countries where there is poor sanitation, particularly Asia/SE Asia and in some countries such as Vietnam >70% of the population are carriers with up to 10%–25% exhibiting symptoms.^4^, ^5^, ^6^

Infections are treated using a combination of antibiotics^2^, ^7^ but often multidrug regimens are required often in combination with proton pump inhibitors lasting for up to 14 days. This approach has often discouraged patient compliance. However, the prevalence of AMR (antimicrobial resistance; notably to clarithromycin and metronidazole^8^) is so high that many infected patients are now considered as having fully resistant infections^9^ and in some cases are unable to access antibiotic therapy.^10^ Clarithromycin has been used around the world in standard triple therapy but clarithromycin‐resistant *H. pylori* isolates have been rapidly increasing worldwide, for example, in China from ~15% in 2000 to ~53% in 2014.^11^ In 2017, clarithromycin‐resistant *H. pylori* was included in the WHO's list of 12 antibiotic‐resistant “priority pathogens” that pose the greatest threat to human health.^12^ Importantly, antimicrobial therapy cannot protect against reinfection, and the rate of reinfection is as high as 15%–30% per year.^13^

The greatest burden of *H. pylori* infection is in developing countries (notably China and SE Asia) where there is a clear link to an increased risk of gastric cancer. It has been suggested that a 10‐year vaccination program might significantly reduce the impact of *H. pylori* infection both with regard to symptoms, gastric cancer, and the associated economic burden of disease management.^14^

Conceptually, a vaccine would best be administered orally to enable the production of secretory IgA (sIgA) in the stomach mucosa preventing colonization.^15^, ^16^, ^17^ However, other mucosal delivery routes (intranasal, rectal) have been successfully used.^15^, ^16^, ^17^ Based on the pathogenesis of *H. pylori*, a number of putative protective antigens have been evaluated including urease (subunits UreA and UreB), flagellar antigens (FlaA and FlaB), cytotoxin‐associated gene A (CagA), vacuolating toxin (VacA), and others.^15^, ^16^, ^17^ Vaccine formulations including subunit vaccines, live vector vaccines, DNA vaccines, and other delivery systems have been evaluated.^15^, ^16^, ^17^ One of major problems with oral immunization is that resulting immunity is weak. Accordingly, adjuvants such as cholera toxin (CT), the closely related heat‐labile toxin (LT) of *E. coli* or the B subunit of CT (CTB) have been extensively evaluated.^18^, ^19^

Although there has been considerable effort in vaccine development, few human studies have demonstrated convincing levels of protective immunity.^16^, ^17^ The one promising exception being a recently described vaccine consisting of an orally‐administered protein formulation comprised of UreB fused to LT.^20^ Despite this there is a case for vaccination where even reduced efficacy might shorten existing treatment regimens and help protect against reinfection.^17^ Here, we have evaluated bacterial spore vaccines using UreA and UreB as putative protective antigens. Using oral delivery spore vaccines induced antigen‐specific mucosal IgA and in a mouse colonization model a 1‐log reduction in stomach colonization was observed. Taken together, the use of spore vaccines could have utility for a prophylactic and potentially therapeutic strategy for reducing the impact of *H. pylori* infection.

## 2 MATERIALS AND METHODS

### 2.1 Strains

*Bacillus subtilis* strain PY79 is a prototrophic laboratory strain. A clinical strain of *H. pylori*, strain HP34, was obtained from the Hospital of the University of Medicine and Pharmacy, Hue University, Vietnam. HP34 was isolated (May 5, 2020) from a 54‐year‐old female patient, peptic ulcer patient, with endoscopy displaying superficial duodenal ulceration, inflammation in the fundus, and antral erosions. The virulence genotypes were shown to be positive to *ure*, *ureB*, *cagA*, and *vacA* (Genbank accession number CP122516). Identity was confirmed by whole genome sequencing. The strain was resistant to clarithromycin but sensitive to tetracycline, metronidazole, amoxicillin, and levofloxacin. *H. pylori* was cultured using either selective Horse Blood Agar (HBA) which was prepared using 4% (w/v) Blood Agar Base No. 2 (Oxoid), supplemented with 8% (w/v) defibrinated horse blood (IVAC, Vietnam), 0.2% (v/v) Skirrow's antibiotic selective supplement (consisting of vancomycin [Sigma], 10 μg/mL; polymyxin B [Sigma], 25 ng/mL; trimethoprim [Sigma], 5 μg/mL; amphotericin B [Sigma], 2.5 μg/mL) and 1% (v/v) sodium lactate (Sigma), or Brain Heart Infusion (BHI) medium (Oxoid) containing 5% (v/v) fetal bovine serum (FBS, Thermofisher Scientific). Incubation was made in a microaerophilic chamber using an Oxoid CampyGen 2.5 L Sachet (5%–7% O_2_, 5%–10% CO_2_, and 85% N_2_) at 37°C, with passaging every 48 h. The strain was preserved in BHI supplemented with 15% (v/v) glycerol at −80°C.

### 2.2 General methods

Methods for *B. subtilis* including preparation of spores and extraction of spore coat proteins are described elsewhere.^21^

### 2.3 Construction of *B. subtilis* spores expressing urease antigens

**FIGURE 1:**
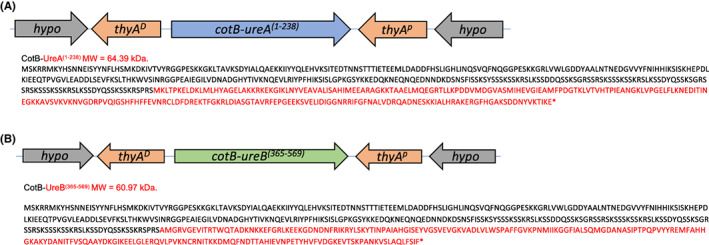
Amino acid sequences of the fusion genes. Chimeric genes inserted at the *thyA* loci (proximal (*thyA* ^*P*^) and distal (*thyB* ^*D*^) segments are indicated) of *B. subtilis* are shown together with a schematic of the chromosomal region (hypo = hypothetical gene). Urease A (*ureA*) and urease B (*ureB*, C‐terminal region) were fused in frame to the 3′‐end of the spore coat protein gene. The MW of the chimeric proteins are shown.

A cloning method referred to as THY‐X‐CISE^®^ ^22^ was used to introduce heterologous genes into the chromosome of *B. subtilis*. First, chimeric genes were synthesized (Azenta Life Sci.) carrying the 5′ segment (including promoter) of the *cotB* gene of *B. subtilis* fused at their 3′‐end to DNA encoding either the full‐length *ureA* gene (encoding UreA, amino acids 1–237; MW 26.4 kDa) or a segment of *ureB* termed *ureB* ^*CT*^ (encoding the non‐enzymatic carboxy‐terminus of UreB,^23^, ^24^ amino acids 365–568, MW 22.9 kDa; Figure 1). The *ureA* and *ureB* sequences were those from *H. pylori* strain 26,695.^25^ The resulting CotB‐UreA and CotB‐UreB^CT^ chimeric polypeptides have predicted MWs of 63 and 61 kDa, respectively. Chimeras were then subcloned into the plasmid pThyA.^22^ This places the chimeric gene between the left (proximal) and right (distal) arms of the *thyA* gene that encodes thymidine synthetase A (Figure 1). Linearization of the resulting plasmid and introduction into the *B. subtilis* chromosome (strain PY79) by a double crossover recombination generates trimethoprim‐resistant transformants that will starve in the absence of thymine (thymine‐dependent). The resulting *thyA::insertion* strain was then used as a recipient in a second transformation where an empty pThyB vector is introduced thus disrupting the *thyB* locus (*thyB::Δ*). The resulting strain (*thyA::insertion thyB::Δ*) is thymine‐dependent but resistant to a higher concentration of trimethoprim. Strains constructed were PK78 *thyA::cotB‐ureB* ^*CT*^ *thyB::Δ* and PK82 *thyA::cotB‐ureA thyB::Δ* referred to henceforth as PK78 (*cotB‐ureB* ^*CT*^) and PK82 (*cotB‐ureA*), respectively. An isogenic strain, PK118 (*thyA::Δ thyB::Δ*), that carries no gene insertions was made using the same procedure with empty pThyA and pThyB vectors that carries no gene insertions and referred to henceforth as PK118 (WT). Strains were confirmed by preparing spores, extracting spore coat proteins, and probing western blots with polyclonal (rabbit) antibodies recognizing UreA (Fisher Sci. Cat No. 17240004) and UreB (Fisher Sci. Cat. No. 17250004).

### 2.4 Detection of surface display of antigens using enzyme‐linked immunosorbent assay

The whole‐spore enzyme‐linked immunosorbent assay (ELISA) protocol was followed as described elsewhere.^26^ Briefly, spores were diluted to 2 × 10^8^ spores/mL in PBS, and 50 μL of suspension was used to coat microplate wells (Greiner, high binding) overnight at 4°C. This was followed by blocking for 1 h at 37°C with PBS containing 0.05% (v/v) Tween 20 and 2% (w/v) bovine serum albumin (BSA). Primary antibodies to the relevant *H. pylori* domains: anti‐UreA raised against the entire UreA polypeptide (Thermo Fisher Sci., PA5‐117505) and anti‐UreB raised against the entire UreB polypeptide (Thermo Fisher Sci., PA5‐32168) were diluted in conjugate buffer (1:2000 in 0.01 M PBS, 1% [w/v] BSA, 1% [v/v] and 0.05% [v/v] Tween 20) and incubated for 2 h at 30°C. The appropriate horseradish peroxidase‐conjugated anti‐rabbit IgG (Sigma Cat No. 12‐348) or anti‐mouse IgG (Dako Cat No. P0447) was diluted in conjugate buffer (1:2000 in 0.01 M PBS, 1% [w/v] BSA, 1% [v/v] and 0.05% [v/v] Tween 20) and used as a secondary antibody. Plates were incubated for 1 h at RT and then developed using tetramethyl benzidine (TMB) substrate (0.1 mg/mL 3.3′,5.5′‐tetramethylbenzidine in 0.1 M sodium acetate buffer [pH 5.5]). Reactions were stopped using 2 M H_2_SO_4_, and ODs read at 450 nm.

### 2.5 rUreA and rUreB^CT^ polypeptides

Recombinant versions of UreA and UreB^CT^ intended for ELISA assays were expressed and purified from *E. coli*. Essentially, PCR was used to amplify DNA fragments encoding open reading frames for UreA (residues 1–237) and UreB^CT^ (residues 365–568) using primer pairs 3080 and 3081 and 3082 and 3083, respectively. The pET‐3d expression vector backbone was amplified using primer pair 522 and 1497. Primer sequences are available upon request. Purified PCR amplicons were assembled using the Klenow Assembly Method and then used to transform *E. coli* Turbo to produce plasmids P90‐pET‐3d‐*UreA* and P91‐pET‐3d‐*ureB* ^*CT*^. Protein expression was conducted using *E. coli* Rosetta cells freshly transformed with either plasmid. Cells were cultured in 400 mL LB medium at 37°C until an A_600_ of approx. 0.8 was attained, and then, the temperature reduced to 16°C for 20 min before inducing protein expression by adding IPTG to 0.2 mM. Expression continued overnight (~18 h) before the cells were harvested, lysed by sonication, and then clarified by centrifugation. Recombinant UreA and UreB^CT^, both of which were designed to be expressed with C‐terminal hexa‐histidine tags, were purified from cell lysates using Ni‐NTA agarose resin (Qiagen) and then buffer exchanged using Amicon Ultra‐4 Centrifugal filters (10 kDa MWCO) into 50 mM Tris–HCl, pH 7.5, containing 50 mM NaCl.

### 2.6 Animal studies

#### 2.6.1 Oral immunizations

Inbred mice (C57 BL/6, females, 9 weeks of age) were used for immunity studies and were housed in groups (*n* = 6). The dosing intra‐gastric (i.g., 0.2 mL) regimen is shown Figure S1A. Groups were Gp1 (naive), dosed with PBS; Gp2, dosed with PK118 (WT) spores; Gp3, dosed with PK78 (*cotB‐ureB* ^*CT*^) spores and Gp4, dosed with PK82 (*cotB‐ureA*) spores. The dosing regimen consisted of four doses with each dose corresponding to three daily administrations (i.g.) of 0.2 mL (PBS or spore vaccine). For Gps 2–4 each i.g. administration consisted of 1 × 10^10^ spore CFU and daily administrations were used to reduce viscosity of the i.g. inoculation. Samples of feces were taken on Days−1, 15, 31, 46, and 61, and serum was taken on Day 62.

#### 2.6.2 Challenge studies

Mice (Mlac:ICR, males, 5–6 weeks of age, 18–20 g) were used for this study. Dosing schedules are shown in Figure S1B and consisted of four oral (i.g.) doses on Days 0, 14, 28, and 53. Four groups (Gp; *n* = 6) were used; Gp1, naive receiving sterile PBS, Gp2, PK118 (WT) spores, Gp3, PK82 (*cotB‐ureA*), and Gp4, PK78 (*cotB‐ureB* ^*CT*^). i.g. dosing consisted of 0.2 mL of either PBS (Gp 1) or spores (1 × 10^10^ CFU; Gps 2–4). On Days 7–9 following the last dose animals were challenged daily with 0.2 mL/day of freshly grown *H. pylori* HP34 culture qualified by OD_600_ measurements to contain ~10^8^ *H. pylori* CFU. Samples of stomach were taken on Day 83 to enumerate *H. pylori* CFU by plating on HBA.

### 2.7 Determination of mucosal titers by indirect enzyme‐linked immunosorbent assay

For analysis of immunological responses, fecal samples were collected on Days−1, 15, 31, 46, and 61 and serum on Day 62 and stored at −80°C. For feces, sample extractions were made at a one‐fifth (w/v) dilution in extraction buffer (2% [v/v] fetal calf serum) containing protease inhibitors, EDTA (0.05 mg/mL), as previously described.^27^ Samples were gently shaken for 2 h at 4°C to disrupt solid material, centrifuged (8000 *g*, 15 min) and the supernatant used for analysis. Antibody levels in feces (IgA) and serum (IgG) were quantified by indirect enzyme‐linked immunosorbent assay (ELISA). Greiner 96‐well plates (MaxiSorp) were coated with 8 μg/mL of rUreA or 4 μg/mL of rUreB^CT^ (50 μL/well) in PBS overnight at 4°C, followed by blocking for 1 h at RT with PBS containing 2% (w/v) bovine serum albumin (BSA). Fecal samples were diluted 1:20 in PBS. Serum samples were diluted 1:10, in diluent buffer (0.01 M PBS, 1% [w/v] BSA, 2% [v/v] FBS, 0.1% [v/v] Triton X‐100, 0.05% [v/v] Tween 20). Samples were added to plates and twofold serially diluted. Plates containing fecal samples were incubated for 2 h at 30°C and those containing serum samples incubated for 2 h at RT. Levels of IgA and IgG were detected using the appropriate horseradish peroxidase‐conjugated anti‐mouse IgA (Sigma Cat No. A4789‐1) or anti‐mouse IgG (Dako Cat No. P0447) in conjugate buffer (2% [v/v] FBS, 1% [v/v] BSA, 0.05% [v/v] Tween 20 in 0.01 PBS). Plates were incubated for 1 h at RT and then developed using tetramethyl benzidine (TMB) substrate (0.1 mg/mL 3.3′,5.5′‐tetramethylbenzidine in 0.1 M sodium acetate buffer [pH 5.5]). Reactions were stopped using 2 M H_2_SO_4_, and ODs read at 450 nm. Dilution curves were created for each sample and endpoint titers estimated as the maximum dilution that gave an absorbance reading above the average naive sample.

### 2.8 Ethics approval

Murine studies were conducted with approval from Royal Holloway University of London Ethics Committee under and an approved UK Home Office animal project license PB9FA6ABB. Challenge studies were conducted with approval from the Research and Ethics Committee of the Institute of Vaccines and Biological Medicals (IVAC; decision no. 241/QD‐VXSPYT 29/07/2022).

### 2.9 Statistical analysis

Statistical significance was assessed by the Mann–Whitney *U*‐test or the Dunnett's test using Prism (GraphPad, Dotmatics).

## 3 RESULTS

### 3.1 Display of urease antigens on the spore coat of *B. subtilis*

The complete urease A protein and the carboxy‐terminus of urease B of *H. pylori* were expressed on the surface of *B. subtilis* spores by in‐frame fusion of the relevant *ureA* and *ureB* coding ORFs to the *B. subtilis cotB* gene (Figure 1; n.b., we were unable to express the complete UreB protein on the spore surface). Both UreA and UreB have been shown to confer protection when delivered orally.^28^, ^29^, ^30^, ^31^ Here, however, we used a truncated urease B that lacked the amino‐terminal enzymatic domain.^23^, ^24^ The *cotB* gene has been used repeatedly for expression of heterologous antigens on *B. subtilis* spores and enables stable presentation of chimeric polypeptides. Our method for cloning used the THY‐X‐CISE® system that places the chimeric genes at the *thyA* (thymidylate synthetase A) gene of the prototrophic *B. subtilis* strain PY79.^22^

**FIGURE 2:**
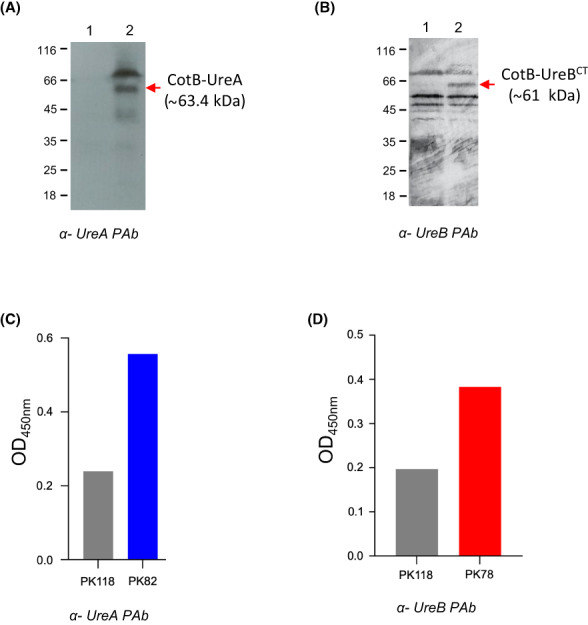
Spore coat expression of urease proteins. *B. subtilis* vaccine strains carrying insertions at the *thyA* loci were examined by western blotting of SDS‐PAGE (12% w/v) size‐fractionated spore coat proteins extracted from preparations of pure spores (approx. 2 × 10^9^ spores/extraction). Blots were probed with PAbs as shown (panels A, B). Surface expression was also determined by whole spore enzyme‐linked immunosorbent assay (ELISA) using microtiter plates coated with spores at 1 × 10^8^ CFU/well (panels C, D). Panel A shows blots of coat proteins extracted from PK82 (cotB‐ureA) and the isogenic parent strain PK118 (WT). Lane 1, spores of the PK118; lane 2, PK82 probed with anti‐UreA PAbs. Bands corresponding to CotB‐UreA (~63.4 kDa) are shown. Panel B shows blots of coat proteins extracted from PK118 (WT) and PK78 (cotB‐ureB^CT^). Lane 1, PK118 spore coat extracts; lane 2, PK78 spore coat extracts. Blots were probed with anti‐UreB PAbs. A band corresponding in size to CotB‐UreB^CT^ and of the correct size (~61 kDa) of the CotB‐UreB^CT^ chimera is indicated. Panel C, PK118 (WT) and PK82 (cotB‐ureA) spores used in ELISA and labeled with anti‐UreA PAbs (1:1000) followed by anti‐rabbit IgG‐HRP secondary antibody (1:3000). Panel D PK118 (WT) and PK78 (cotB‐ureB^CT^) spores used in ELISA and labeled with anti‐UreB PAbs (1:2000) followed by anti‐rabbit IgG‐HRP secondary antibody (1:3000).

This was followed by insertional disruption of the *thyB* locus resulting in strains (PK82 *cotB‐ureA* and PK78 *cotB‐ureB* ^*CT*^) that are unable to grow in the absence of thymine (or thymidine). Using a congenic strain (PK118) that carried insertional disruption of both *thyA* and *thyB* but carried no chimeric genes two immunological methods were used to verify surface display. First, western blotting using PAbs that recognize UreA and UreB and second, whole spore ELISA (Figure 2).

Whole spore ELISA clearly demonstrated recognition of UreA and UreB on spores with some cross‐reaction to PK118 spores (Figure 2C,D). It should be noted that a tricistronic urease operon (*ureABC*) is present in most strains of *B. subtilis* with *ureC* corresponding to the enzymatic subunit that in *H. pylori* is named *ureB*.^32^ *B. subtilis* UreA shares some homology with *H. pylori* UreA (~31%) and with UreC about 75% homology with *H. pylori* UreB. The operon is transcribed during ordinary vegetative cell growth but only at high levels during nitrogen‐limited growth.^32^, ^33^ Using a standard agar‐based biochemical method (Christensen's slant agar^34^), we have confirmed that all three strains (PK118, PK78, and PK82) do not produce functional urease (not shown). Although we produced crops of spores, we cannot, however, rule out the possibility of the presence of low levels of *B. subtilis*‐produced urease being present (possibly adsorbed to spores) and possibly accounting for this cross‐reaction.

Blotting of size‐fractionated spore coat extracts for PK82 (*cotB‐ureA*) revealed three bands (~40, [diffuse], 64 and 70 kDa.) that were absent in PK118 spores (Figure 2A,B). One of these bands was in close agreement with the predicted size (63.4 kDa) of the CotB‐UreA chimera (Figure 2A; the other bands most likely being multimeric or breakdown species). Western blotting of PK78 (*cotB‐ureB* ^*CT*^) was less clean with cross‐reacting bands in PK118 spores. However, one abundant band of the correct size for CotB‐UreB (~61 kDa) was clearly present in PK78 and absent in PK118 spores (Figure 2B). Since the cross‐reacting bands are associated with the spore coat the most likely explanation is that of cross‐recognition with *B. subtilis* spore coat proteins. However, we were unable to identify any spore coat protein that showed significant levels of amino acid homology with either urease A or B.

### 3.2 Immune responses in mice dosed with spore vaccines

Mice were dosed orally (i.g.) four times with spores of PK82 (CotB‐UreA), PK78 (CotB‐UreB^CT^) and the isogenic control PK118 strain that expressed no heterologous polypeptides. To enable a dose of 3 × 10^10^ spore CFU three daily administrations of 1 × 10^10^ spores were required since high concentrations of spores in suspension are typically overly viscous. A naive group receiving only PBS provided a baseline.

**FIGURE 3:**
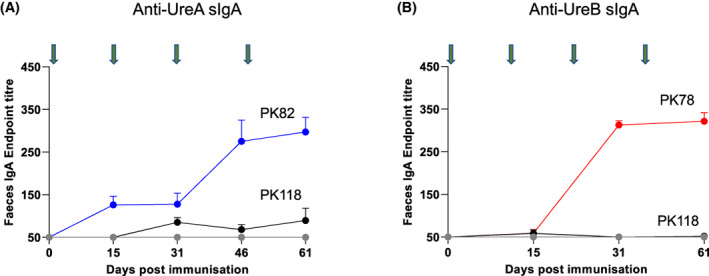
Mucosal responses following oral administration of spore vaccines expressing *H. pylori* antigens. Mice (C57 BL/6) were dosed (i.g.) with spores of PK118 (WT), PK82 (CotB‐UreA), or PK78 (CotB‐UreB^CT^) four times (green arrows). Each dose comprised three separate administrations (1 × 10^10^ CFU/administration); dose 1 (Days 1–3), dose 2 (Days 16–18), dose 3 (Days 32–34), and dose 4 (Days 47–49). rUreA‐specific (panel A) and rUreB‐specific sIgA (panel B) in longitudinal fecal samples in fecal samples from PK82, PK78, or PK118 dosed mice are shown.

Measurement of antigen‐specific sIgA in fecal samples showed seroconversion to both UreA (PK82‐dosed) and UreB (PK78‐dosed; Figure 3). Maximal antibody responses were observed at Day 61. Responses for both PK78 and PK82‐dosed animals, at maximum, were significantly (*p* = 0.0001) greater than in mice dosed with PK118 spores or the naive group. Very low levels of UreA‐specific sIgA were observed in PK118‐dosed mice but these were not statistically significant.

**FIGURE 4:**
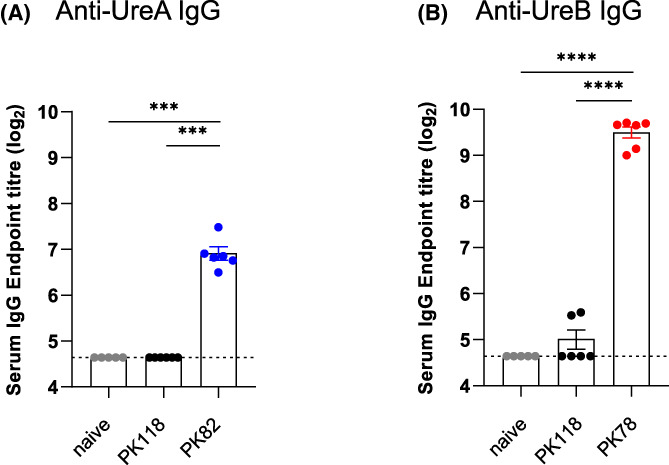
Systemic responses following oral administration of spore vaccines expressing *H. pylori* antigens. Mice (C57 BL/6) were dosed (i.g.) with spores of PK118 (WT), PK82 (*CotB‐UreA*), or PK78 (*CotB‐UreB* ^*CT*^) four times. Each dose comprised three separate administrations (1 × 10^10^ CFU/administration); dose 1 (Days 1–3), dose 2 (Days 16–18), dose 3 (Days 32–34), and dose 4 (Days 47–49). rUreA‐specific (panel A) and rUreB‐specific (panel B) IgG in serum samples taken at Day 61 are shown. Mann–Whitney, ***p = 0.001, ****p = 0.0001.

Serum IgG responses measured at Day 61 also showed that both PK78 and PK82 were able to induce systemic immunity (Figure 4). Taken together, oral administration of spores expressing either UreA or UreB^CT^ on the spore surface can elicit both mucosal and systemic responses.

### 3.3 Protection in a murine colonization model

**FIGURE 5:**
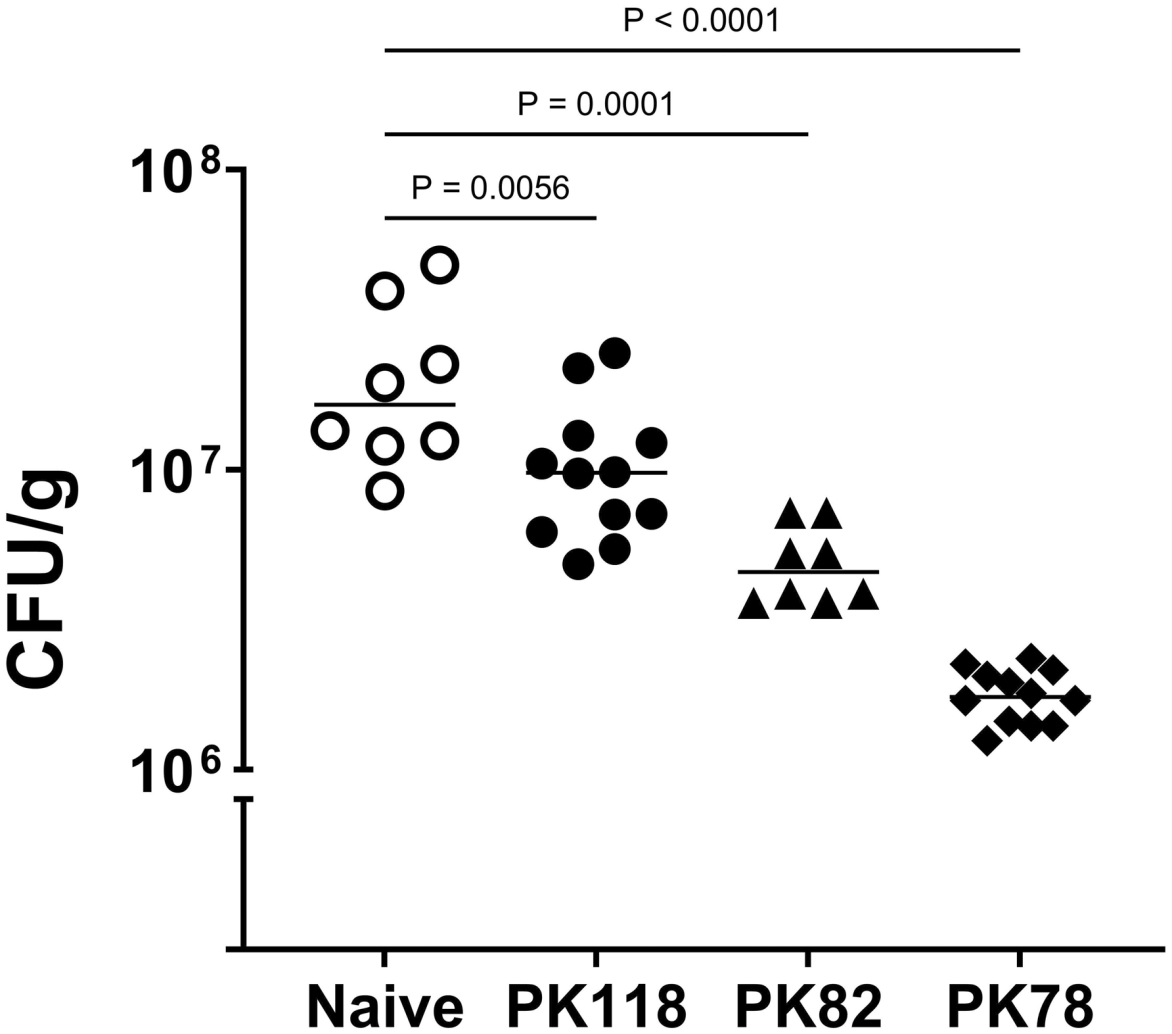
*H. pylori* colonization in immunized mice. Bacterial loads of *H. pylori* HP34 in stomach samples 21‐day post‐challenge. Animals had been orally (i.g.) dosed four times with spores (1 × 10^10^ CFU/dose) of PK118 (WT), PK82 (*CotB‐UreA*), or PK78 (*CotB‐UreB* ^*CT*^) and challenged 7–9 days after the last immunization. Naive mice received PBS. The data combine samples from two independent repeat studies with *p* values as shown.

Mice were given four oral (i.g.) doses of spores (1 × 10^10^/dose) of either PK118 (WT), PK78 (CotB‐UreB^CT^) or PK82 (CotB‐UreA), as well as a naive group, and then challenged with *H. pylori* (i.g.) using a challenge dose of ~10^8^ CFU. Stomach samples were taken 21 days post‐challenge for enumeration of *H. pylori* CFU. The study was repeated, and combined CFU data are shown in Figure 5.

PK78 (CotB‐UreB^CT^) immunized animals showed the greatest reduction in *H. pylori* CFU of about 1‐log. PK82 (CotB‐UreA) dosed animals also showed a significant reduction (~72%, median values; *p* = 0.0001) in CFU but less so than PK78 dosed animals (89%, median values, *p* < 0.0001). Interestingly, animals dosed with “naked” spores (PK118), that is, spores displaying no *H. pylori* antigens, also showed a reduction (~40%, median, *p* < 0.01) in *H. pylori* CFU compared to naive animals. In conclusion, both spore vaccines expressing either UreA or UreB^CT^ were able to confer protective immunity sufficient to reduce *H. pylori* colonization in mice.

## 4 DISCUSSION

Spores of *B. subtilis* have been used extensively as mucosal vaccine vectors where oral (intra‐gastric or sublingual) or nasal administration efficiently induces mucosal immunity (typically sIgA) as well as a Th1 bias.^35^, ^36^, ^37^ Antigens are displayed on the spore surface (diameter ~ 1 μm) typically fused (as chimeric fusions) to proteins associated with the outermost layers of the spore coat. *Bacillus* spores are dormant yet able to germinate and outgrow under favorable conditions. They are also particularly robust being able to survive exposure to extremes of heat, desiccation as well as noxious compounds including gastric fluids.^38^ Remarkably, and as a rule, chimeric spore expression does not normally lead to significant degradation of the exposed heterologous protein. Spores are members of the aerobiome and also found in soil and vegetation.^39^, ^40^ As such animals and humans are exposed to a low level of *Bacillus* on a daily basis.^41^, ^42^ Considered together, the use of bacterial spores as oral vaccine vehicles is compelling.

For *H. pylori* vaccination, we evaluated two “classical” antigens, UreA (urease A) and UreB (urease B) since these have been used extensively in vaccine formulations and in animal studies show evidence of protection.^29^, ^31^, ^43^, ^44^, ^45^ Our data show firstly that 12 oral administrations of spores expressing either UreA or UreB^CT^ evoked mucosal immunity evident from seroconversion of antigen‐specific sIgA in fecal samples although it should be noted that IgG is also present in mucosal samples^46^ and we did not assess levels of this immunoglobulin. UreB has previously been used for *H. pylori* vaccination utilizing spores for oral delivery but this has incorporated the entire UreB polypeptide fused to the CotC spore coat anchor.^28^, ^47^ Here, we chose a truncated UreB domain, UreB^CT^, to optimize spore expression since the use of the CotB anchor partner significantly increases the size of the resulting hybrid protein. Secondly, systemic antigen‐specific IgG responses were also induced. Finally, when mice were dosed with UreA or UreB^CT^ spores and then challenged with *H. pylori* the resulting counts of *H. pylori* CFU in the stomach were reduced by about 1‐log (for CotB‐UreB^CT^). These data are broadly similar to those obtained by Zhou et al.^28^ using spores expressing the full‐length UreB protein (CotC‐UreB) although there were some differences that need discussion. First, Zhou et al.^28^ evaluated, in parallel, spores of an isogenic control strain that did not express any *H. pylori* antigens and in protection studies mice showed no reduction in counts of *H. pylori*. This is in marked contrast to our work here which showed that spores alone (i.e., PK118 spores) conferred a low level of protection (40% reduction in gastric CFU). These spores do not evoke antigen‐specific sIgA so the most probable explanation is that of innate immunity. *Bacillus* spores have been well documented as being able to evoke innate immunity and for some pathogens such as influenza this can be protective.^48^, ^49^, ^50^ This has included murine studies showing reduced colonization by *Clostridium difficile* following oral dosing with “naked” spores.^35^ We suspect that repeat dosing with *B. subtilis* spores may trigger an innate immune response sufficient to exert some level of protection. A second point is that Zhou et al.^28^ also evaluated a trimeric fusion protein comprising a CotC anchor fused to CTB (cholera toxin subunit B) and UreB. This vaccine provided the highest reduction in gastric CFU of ~90% and was thus similar to our data found here for CotB‐UreB^CT^.^28^ CTB was employed as a mucosal adjuvant but we suspect that the natural and well documented microparticulate adjuvant properties of spores are sufficient to provide adjuvancy dispensing with the need for an auxiliary mucosal adjuvant.^50^, ^51^

Here, we evaluated two different antigens and neither have been previously evaluated using spores (note that Zhou et al. used complete UreB^28^). Examination of other in vivo studies on *H. pylori* live‐vectored vaccines reveals that 90% reduction in *H. pylori* CFU is close to the maximum that can be achieved. That both UreA and UreB delivered on spores confers some level of protection reinforces the general observation that a variety of *H. pylori* antigens can be used for vaccination.^17^ It also supports the notion that other elements of the immune system may be required to achieve full sterilizing immunity.^15^, ^52^ It is well documented that cell‐mediated immunity plays an important role in protection against *H. pylori* infection.^15^, ^16^ Gastric biopsy samples from infected patients display an increase in CD4+ *T* cells,^52^ and a bias of Th1 cells has been considered necessary for protection.^53^ In addition, UreB has been shown to induce Th17 cells that, in turn, are responsible for the production of the proinflammatory cytokines, IL‐17, IL‐17F, and IL‐22.^54^ Oral administration of *Bacillus* spores has been shown in mice to interact with components of the cellular immune system, notably toll‐like receptors (TLRs), with in vivo induction of proinflammatory cytokines (TNF‐α and IL‐6).^55^ Potentially, these phenotypes may be linked with the abovementioned innate response but we suspect that the immunostimulatory properties of spores alone may also be contributing to the inhibition of *H. pylori* colonization. Lastly, as humans are exposed to low levels of *Bacillus* on a daily basis future development of the spore platform must consider and address the issue of tolerance and suppression of the immune response.^56^

## 5 CONCLUSION

This work has shown the potential utility of spores for prophylactic vaccination to *H. pylori* infection. The use of a system that ensures containment of genetically modified probiotic spores is a further advantage primarily because the use of GMOs in humans remains contentious and biological containment using the approach reported here is assured. A therapeutic application of a *H. pylori* spore vaccine is also worthy of consideration and is under current investigation. Finally, the spore platform enables other potential *H. pylori* antigens to be evaluated and potentially a multivalent vaccine to be formulated. Such an approach might further boost levels of protection.

## AUTHOR CONTRIBUTIONS

PMK, TLPN, TKCN, GM, and DMDB conducted experimental studies. VDN, GC, HAH, and SMC designed studies. SMC wrote the manuscript.

## Supporting information


Figure S1.

## Data Availability

The data that support the findings of this study are available from the corresponding author upon reasonable request.
