# Supplementary material for: Prophylactic immunization to Helicobacter pylori infection using spore vectored vaccines
Source: Helicobacter. 2023 Jun 14;28(4):e12997. doi: 10.1111/hel.12997 (PMC10909515; doi:10.1111/hel.12997)
Supplement: Supplementary file 1 — Figure S1. [file HEL-28-e12997-s001.pdf]

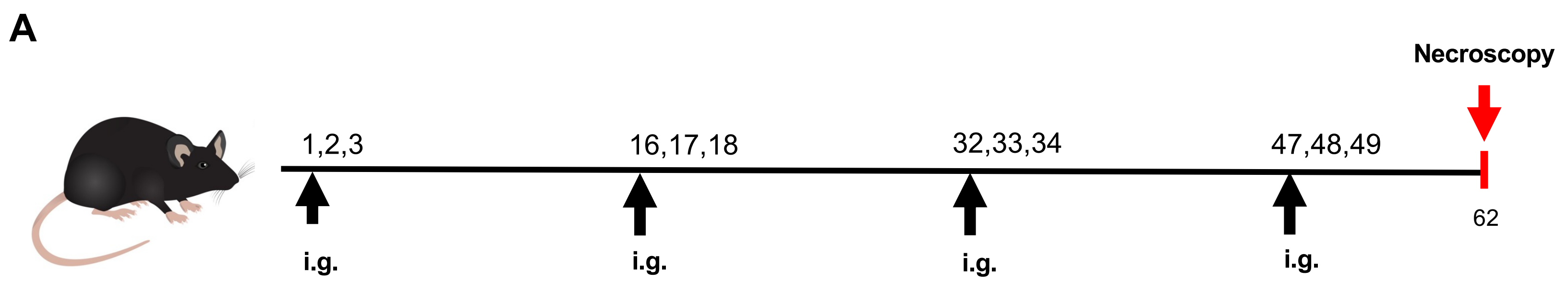

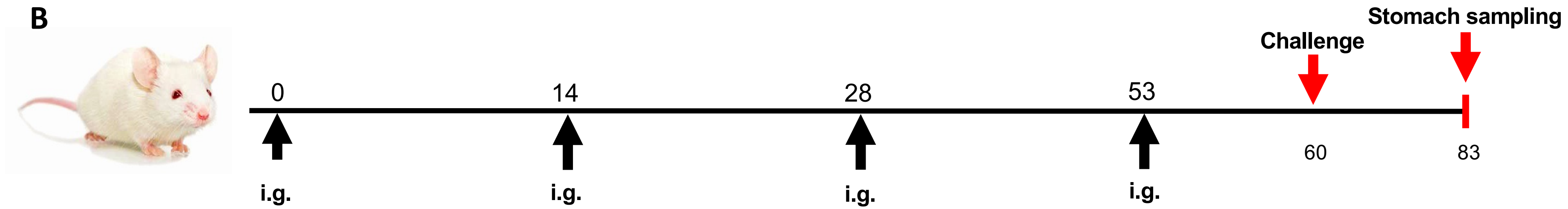

<u>Supplementary Figure 1: Animal Study Schedule</u>

A) Immune Response Study: Mice (C57 BL/6) were immunised by the oral route (intra-gastric, i.g.) with suspensions of either spores expressing CotB-UreA or CotB-UreB^{CT} or control spores (wild type PY79). A naive, non-immunized control group was included. Oral immunizations were performed with 1.0 × 10^{10} spores in a volume of 0.2 ml of water administered by intragastric lavage on days 1, 2, 3, 16, 17, 18, 32, 33, 34, 47, 48 and 49. Animals were sacrificed on day 62, and serum samples were collected (heart bleed). Faeces where collected on days -1, 15, 31, 46 and 61.

B) Challenge Study: Mice (Mlac:ICR) were immunized by the oral route (i.g.) or sublingual (s.l.) route with suspensions of either spores expressing CotB-UreA, CotB-UreB^{CT} or PK118 (control spores). A naive, non-immunized control group was also included. Oral immunizations were performed with a volume of 0.2 ml of 1.0 × 10^{10} spores on days 0, 14, 28, and 53. Mice were then challenged with *H. pylori* HP34 on days 60. Samples of stomach were taken on day 83 to enumerate *H. pylori* CFU.
